# Emerging Regulatory Mechanisms in Sinoatrial Node Automaticity

**DOI:** 10.1111/jcmm.71058

**Published:** 2026-02-13

**Authors:** Hongyu Liu, Yuting Cao, Xuling Su

**Affiliations:** ^1^ Department of Pathology Shanghai Sixth People's Hospital Affiliated to Shanghai Jiao Tong University School of Medicine Shanghai China; ^2^ Department of Rehabilitation Medicine Shanghai Sixth People’s Hospital Affiliated to Shanghai Jiao Tong University School of Medicine Shanghai China; ^3^ Department of Laboratory Diagnostics Third Affiliated Hospital of Naval Medical University (Eastern Hepatobiliary Surgery Hospital) Shanghai China

**Keywords:** coupled‐clock theory, glutamatergic signalling, molecular modulators, pacemaker cell microenvironment, sinoatrial node automaticity

## Abstract

The sinoatrial node (SAN), the primary cardiac pacemaker, governs rhythmic heartbeats through spontaneous electrical impulses. While the classical “coupled‐clock” theory, integrating the membrane voltage clock (driven by cyclic ion channel activity) and the calcium clock (orchestrated by rhythmic sarcoplasmic reticulum Ca^2+^ release), remains central to understanding pacemaker automaticity, recent research has unveiled multifaceted regulatory mechanisms that may complement this core model. This review synthesises current evidence on the critical roles of pacemaker cell‐microenvironment interaction, glutamatergic signalling via mitochondrial reactive oxygen species (ROS)‐Ca^2+^ coupling, and novel molecular modulators such as CIRP, SGO1, and GLP‐1. These insights reveal a highly integrated and dynamic regulatory network that potentially modulates SAN automaticity under physiological and pathological conditions. Elucidating these mechanisms not only deepens our understanding of cardiac pacemaking but also identifies potential therapeutic targets for SAN dysfunction and associated arrhythmias.

## 1 Introduction

The sinoatrial node (SAN) is the primary pacemaker of the heart, initiating electrical impulses that propagate through the atria to the atrioventricular node and subsequently through the His‐Purkinje system to the ventricles, thereby orchestrating cardiac contractions and ensuring efficient blood circulation throughout the body [1, 2]. Despite its critical role in cardiac conduction, the SAN remains a relatively poorly understood cardiac structure due to its small size, complex composition, and intricate functional mechanisms [2]. Consequently, elucidating the mechanisms governing SAN automaticity is essential for treating SAN dysfunction, a relatively common clinical condition that manifests as bradycardia [3], sinus arrest or block [4], and increased susceptibility to atrial fibrillation [5].

SAN automaticity is fundamentally dependent on the interplay between the membrane clock and the calcium clock, which together form the basis of the coupled‐clock theory of cardiac pacemaking [6, 7, 8, 9]. The membrane clock is composed of cell‐surface ion channels, including the hyperpolarisation‐activated cyclic nucleotide‐gated (HCN) current (*I*_f_) [10], L‐type and T‐type calcium current (*I*_Ca,L_ and *I*_Ca,T_) [11], and the electrogenic sodium‐calcium exchanger (*I*_NCX_) [12]. The calcium clock, on the other hand, is governed by intracellular calcium cycling, primarily through sarcoplasmic reticulum (SR) calcium release via ryanodine receptors (RyRs). This calcium release drives local calcium release (LCR) events, which in turn trigger spontaneous firing in SAN pacemaker cells (PCs) [11]. The sequential and cyclic activation of the membrane clock and the calcium clock ensure the automaticity and rhythmicity of PCs and the generation of a stable heart rate [13, 14].

SAN plasticity refers to the ability of the SAN to adapt and change its structure and function in response to various physiological and pathological stimuli. Plasticity in the SAN can be driven by factors such as autonomic nervous system (ANS) activity. The ANS regulates SAN function by modulating the balance between sympathetic and parasympathetic inputs, thereby controlling PCs automaticity through the membrane and calcium clocks [15, 16]. This regulation is largely mediated by the activation of adenylyl cyclase (AC) in PCs [14, 17, 18]. Sympathetic stimulation, via AC activation, increases cytosolic cyclic adenosine monophosphate (cAMP) levels, accelerating diastolic depolarisation and increasing firing rate through HCN4 and RyR‐mediated local subsarcolemmal calcium releases [14]. Conversely, parasympathetic stimulation, mediated by muscarinic receptors, reduces SAN automaticity by decreasing cAMP levels and inhibiting AC activity. The crosstalk between autonomic modulation and the coupled‐clock system is thus pivotal for maintaining SAN automaticity.

While the interplay between the ANS and the coupled‐clock theory provides a foundational understanding of SAN automaticity [7, 9], recent research has unveiled new mechanisms underlying SAN pacemaker activity. This review focuses on recent advances in understanding SAN automaticity, with particular emphasis on the PCs microenvironment [19, 20], glutamatergic signalling, and calcium dynamics [21, 22, 23], key molecular regulators (e.g., CIRP, SGO1, and GLP‐1) [24, 25, 26]. By synthesising recent research findings, this review aims to provide a comprehensive overview of these novel mechanisms.

## 2 Architectural and Functional Complexity of the Sinoatrial Node

**FIGURE 1:**
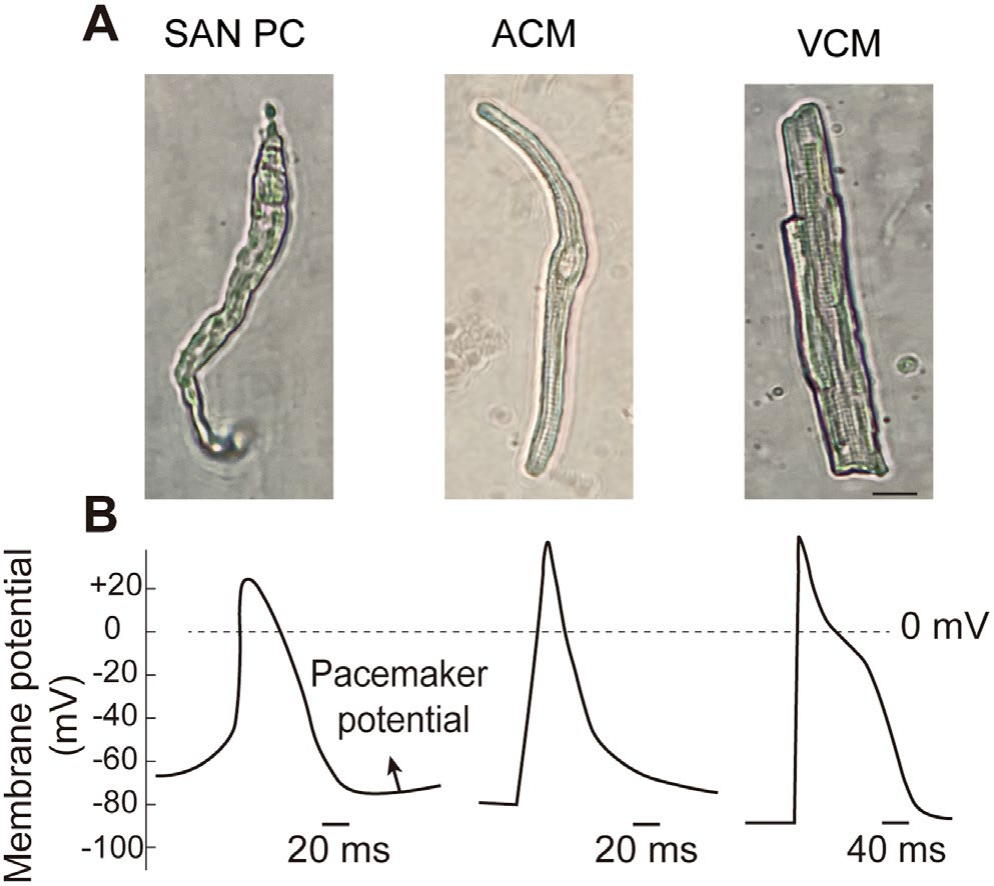
The morphological and action potential characteristics of mouse SAN pacemaker cells (SAN PCs) and working cardiomyocytes. (A) SAN PCs are distinguished morphologically from working cardiomyocytes. These specialised cells are long, thin cells with few striations. (B) The APs of PCs are markedly different from those of the working cardiomyocytes (ACMs and VCMs), characterised by diastolic phase 4 depolarisation, also known as the “pacemaker potential”. The scale bar = 10 μm. The cell isolation and APs recording of SAN PCs, ACMs, and VCMs were performed as our previously described [21, 32]. ACM, atrial cardiomyocyte; VCM, ventricular cardiomyocyte.

The structure and composition of the mammalian SAN have been well characterised [27]. The SAN is a crescent‐shaped structure located at the junction of the superior vena cava and the right atrium, along with the crista terminalis [2, 28]. The SAN is primarily supplied by the sinoatrial nodal artery, a branch of the right coronary artery [29]. At the SAN core, a distinct population of spindle‐shaped leading PCs generates spontaneous electrical impulses, distinguishing them functionally and morphologically from working cardiomyocytes [30]. These specialised cells lack intercalated discs and exhibit reduced cell size to minimise electrotonic coupling to surrounding atrial tissue [31] (Figure 1A). Notably, the dominant pacemaker site exhibits dynamic positional plasticity, shifting in response to autonomic stimuli (sympathetic/vagal activation), pharmacological interventions, mechanical stretch, and thermal fluctuations [31].

The SAN is a highly complex and heterogeneous tissue composed of multiple cell types, including pacemaker cardiomyocytes, fibroblasts, endothelial cells, immune cells, neurons, and adipose cells [1, 2, 33, 34, 35, 36]. This cellular heterogeneity is characterised by the differential distribution of gap junctions, which facilitate electrical coupling between cardiomyocytes and the propagation of action potentials (APs). Specifically, electrical coupling is weaker in SAN PCs compared to the surrounding atrial myocardium due to high expression of connexin45 (Cx45) and low or absent expression of Cx43 [37, 38, 39]. This unique distribution of gap junctions allows the heterogeneous SAN to synchronise and drive atrial muscle contractions.

Recent research has drawn parallels between the SAN and the brain's neuronal networks, leading to the concept of the SAN as the heart's “central brain” [40]. High‐resolution imaging techniques have revealed the three‐dimensional cytoarchitecture of the mouse SAN, including autonomic innervation, peripheral glial cells, and PCs [40]. These findings are further characterised and discussed in the editorial by Santana et al. [41], which highlights a heterogeneous pattern of autonomic innervation, a web of S100B^+^/GFAP^+^ peripheral glial cells, and a distinct population of S100B^+^/GFAP^−^ interstitial cells interacting with the HCN4‐expressing PCs. This study suggests a neuromimetic microenvironment dedicated to regulating LCR events and intercellular communication among PCs. Furthermore, SAN PCs exhibit glutamatergic neuron‐like properties, sharing molecular and cellular features with glutamatergic neurons, including the expression of glutamate receptors, transporters, and cell markers such as SNAP25 and Slc17a7 [22]. The glutamatergic neurotransmitter system in SAN PCs may serve as an intrinsic regulation system for spontaneous bioelectrical activity. Future research is needed to elucidate the specific mechanisms through which this glutamatergic system interacts with established pacemaking pathways, such as the coupled‐clock model, to refine our understanding of SAN physiology and its potential therapeutic implications.

## 3 The Classical Coupled‐Clock System in SAN Automaticity

The membrane clock, a key component of the coupled‐clock system, is characterised by the unique electrophysiological properties of SAN APs. These APs are markedly different from those of the working atrial myocardium, characterised by diastolic phase 4 depolarisation, also known as the “pacemaker potential” (Figure 1B). The *I*_Ca,L_ is essential for generating rhythmic spontaneous APs, as it provides the inward current necessary for AP upstroke and diastolic depolarisation [11, 35, 42]. Delayed rectifier potassium currents (*I*_k_) facilitate repolarisation, while the absence of inward rectifier potassium current (*I*_ki_) in the SAN allows membrane repolarisation below the threshold for the *I*_f_ current. The *I*_f_ current is a mixed sodium and potassium current that triggers spontaneous and repetitive diastolic depolarisation and activates *I*_Ca,L_ within the SAN. Other currents, including the *I*_Ca,T_ and *I*_NCX_, also contribute to SAN pacemaker activity. Mutations in genes encoding these ion channels, such as SCN5A, calcium channels, and HCN4, result in SAN dysfunction [35, 43]. Additionally, the expression of Nav1.5 increases from the central to peripheral SAN and further in the working atrial myocardium, contributing to the progressive increase in AP upstroke velocity [35]. The synergistic interplay between these ion channels forms the basis of the membrane clock, which is dynamically coupled with the calcium clock to generate robust and rhythmic pacemaker activity.

**FIGURE 2:**
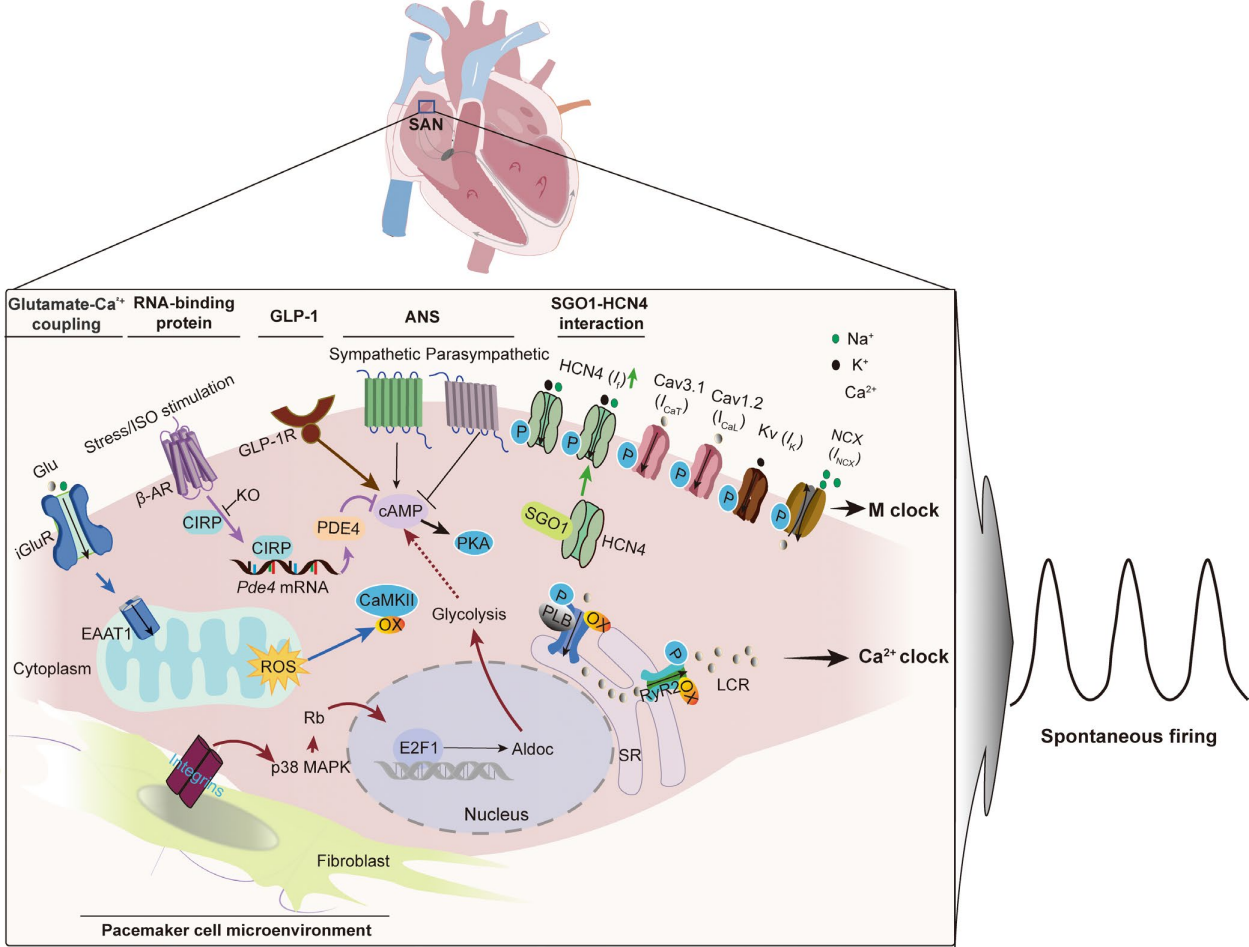
Regulatory network governing SAN automaticity. This schematic depicts the coupled‐clock system underlying sinoatrial node (SAN) pacemaking. The synergistic coupling between the membrane voltage clock (M clock) and the calcium clock (Ca^2+^ clock) generates spontaneous depolarisations, which are modulated by pacemaker cell‐microenvironment crosstalk, glutamatergic signalling, and molecular regulators. SAN pacemaker cells‐fibroblast crosstalk promotes aerobic glycolysis via aldolase C (Aldoc), while glutamatergic signalling enhances local calcium releases (LCRs) through mitochondrial reactive oxygen species (ROS)‐Ca^2+^ coupling, leading to the activation of oxidised Ca^2+^/calmodulin‐dependent protein kinase II (Ox‐CaMKII). Several key molecular regulators include cold‐inducible RNA‐binding protein (CIRP), which under stress prevents pathological cAMP accumulation; Shugoshin‐1 (SGO1), stabilising HCN4 channels to augment the hyperpolarisation‐activated current (*I*_f_) current; and glucagon‐like peptide‐1 receptor (GLP‐1R) signalling, which potentiates *I*_f_ via cAMP/PKA‐dependent HCN4 phosphorylation. ANS, autonomic nervous system; EAAT1, excitatory amino acid transporter 1; Glu, glutamate; *I*_Ca,L_ and *I*_Ca,T_, L‐type and T‐type calcium currents; iGluR, ionotropic glutamate receptor; NCX, sodium‐calcium exchanger; PKA, cAMP‐dependent protein kinase; PLB, phospholamban; RyR, ryanodine receptor; SR, sarcoplasmic reticulum.

The SAN employs intricate regulatory networks to maintain robust automaticity. SAN PCs generate rhythmic APs through the synergistic interplay of two oscillatory systems: the membrane clock (ion channel‐driven depolarisation) and the calcium clock (sarcoplasmic reticulum Ca^2+^ cycling) (Figure 2). The membrane clock relies on phase 4 diastolic depolarisation mediated by the *I*_f_, *I*_Ca,T_, and electrogenic *I*_NCX_. Concurrently, the calcium clock generates rhythmic subsarcolemmal Ca^2+^ releases via RyRs, which activate *I*_NCX_ to further depolarise the membrane. These clocks are dynamically coupled through cAMP/PKA and CaMKII signalling, enabling rapid adaptation to autonomic modulation. Sympathetic stimulation enhances both clocks via β‐adrenergic receptor‐driven cAMP elevation, accelerating heart rate, while parasympathetic input suppresses pacemaking through acetylcholine‐mediated cAMP reduction.

## 4 Emerging Regulatory Mechanisms

While the classical coupled‐clock system integrating the membrane clock and calcium clock remains foundational [6, 8, 9], emerging evidence has uncovered novel regulatory mechanisms that complement this theory.

### 4.1 Fibroblast‐Pacemaker Cell Interactions and Metabolic Reprogramming

The SAN exhibits a specialised extracellular matrix (ECM) enriched with collagen fibres and fibroblasts, forming a unique structural and functional niche for PCs [19, 44, 45]. A defining feature of the mature SAN is its heterogeneous cellular composition, where disorganised PCs are interspersed with non‐pacemaker cells (NPCs), including atrial myocytes, adipocytes, fibroblasts, and dense collagenous networks [36, 45, 46]. These NPCs and ECM components collectively establish a dynamic microenvironment critical for maintaining normal SAN automaticity. Emerging evidence highlights bidirectional interactions between PCs and their microenvironment as essential regulators of pacemaker activity (Figure 2) [19].

Fibroblasts, the most abundant cardiac cell type, interact with PCs in the SAN through integrin‐mediated cell–cell contact [19]. This physical interaction initiates intracellular signalling cascades that induce metabolic reprogramming of PCs, particularly enhancing glycolytic flux [19]. The SAN ECM, characterised by elevated hyaluronic acid, tenascins, and proteoglycans, establishes a mechanically compliant substrate distinct from the stiffer atrial myocardium [47, 48, 49]. Mechanosensitive studies demonstrate that PC cultures on rigid substrates exhibit disrupted subcellular organisation of ion channels (e.g., HCN4) and impaired electrochemical oscillations, underscoring the mechanoelectrical coupling essential for automaticity [47, 48, 49]. Notably, the soft ECM biomechanics optimise intercellular coupling through connexin‐mediated electrotonic interactions while maintaining PC electrophysiological autonomy [47, 48, 49].

Fibroblast‐PC crosstalk drives metabolic remodelling via induction of aldolase C (Aldoc), a glycolytic rate‐limiting enzyme [19, 20, 45]. Specifically, Chou et al. demonstrated that this contact activates integrin β1 on PCs, triggering an intracellular signalling cascade through p38 mitogen‐activated protein kinase (MAPK) [19]. Activated p38 MAPK promotes the dissociation of Rb from E2F1, enhancing E2F1 transcriptional activity [19]. E2F1 then binds to the promoter of Aldoc, upregulating its expression in PCs [19]. Aldoc upregulation enhances aerobic glycolysis, which correlates with increased PC firing rates. Genetic ablation of fibroblast‐PC interactions or Aldoc knockdown abolishes spontaneous electrical activity, confirming the necessity of this metabolic shift for pacemaker function [19, 20, 45]. Beyond metabolic regulation, fibroblasts may facilitate ephaptic conduction, a form of electrical communication between cells that occurs not via gap junctions but through electric fields within narrow extracellular cleft [50]. This microenvironment‐related regulation supplements the traditional coupled‐clock model by introducing external factors that can influence the internal clock mechanisms.

### 4.2 Glutamatergic Signalling and Calcium Clock Amplification

The glutamatergic system, classically associated with central neurotransmission [51, 52, 53, 54], exhibits functional expression in cardiac PCs [21, 22, 23, 55]. SAN PCs express ionotropic glutamate receptors (iGluRs) and metabotropic receptors, enabling autocrine/paracrine regulation of automaticity [22]. Experimental evidence demonstrates that glutamate potentiates spontaneous firing through enhanced SR Ca^2+^ cycling, specifically by amplifying LCRs during diastolic depolarisation (Figure 2) [21].

Mechanistically, mitochondrial excitatory amino acid transporter 1 (EAAT1) mediates glutamate import into PC mitochondria, triggering reactive oxygen species (ROS) elevated generation [21]. ROS function as key secondary messengers that directly couple mitochondrial glutamate signalling to the two clocks. The oxidative modification of Ca^2+^‐handling proteins, including RyR2 (increased open probability), SERCA2a (enhanced uptake kinetics), and CaMKII (sustaining its activation and thereby promoting phosphorylation of downstream targets), synergistically augments LCR magnitude and frequency (Figure 2) [21]. This concerted action critically couples the two clocks: enhanced LCRs (calcium clock) facilitate depolarisation by activating *I*_NCX_, which in turn promotes membrane clock activation; concurrently, ROS‐and CaMKII‐dependent signalling can modulate the activity of membrane ion channels (e.g., facilitating HCN channels) [21]. Genetic EAAT1 depletion reduces SAN firing rates in vitro and blunts physiological heart rate responses in vivo, establishing EAAT1 as a critical regulator of coupled‐clock dynamics [21]. Pharmacological iGluR inhibition similarly suppresses automaticity, suggesting therapeutic potential in arrhythmia management.

In neurons, glutamatergic signalling is mainly involved in neurotransmission between cells [53]. In SAN PCs, while glutamate also serves as a signalling molecule, its primary role is to modulate pacemaker activity at the single‐cell level. Here, the glutamatergic‐ROS cascade establishes a tightly coupled regulatory loop between the clocks [21]. The ROS‐mediated enhancement of LCRs directly accelerates the depolarisation rate via *I*_NCX_, subsequently influencing the membrane clock's activation [21]. Conversely, the membrane potential and calcium influx can influence mitochondrial function and ROS production, creating a potential feedback loop. This glutamatergic‐Ca^2+^‐ROS coupling mechanism adds a new dimension to the traditional coupled‐clock system, positioning glutamate as an LCR “ignition” signal in SAN PCs.

### 4.3 Novel Molecular Modulators: Integrating Stress Response, Genomic Stabilisers, and Metabolic Signalling

The intrinsic automaticity of the SAN is dynamically regulated by a network of molecular mechanisms that integrate electrophysiological, calcium cycling, and metabolic signals. Beyond the established roles of stress‐responsive proteins such as cold‐inducible RNA‐binding protein (CIRP) and shugoshin‐1 (SGO1), emerging evidence highlights the involvement of metabolic and neurohormonal modulators, including glucagon‐like peptide‐1 (GLP‐1), in modulating SAN automaticity. During β‐adrenergic stress, CIRP prevents excessive tachycardia by binding to and stabilising phosphodiesterase 4 (*Pde4*) mRNA, thereby preventing pathological cAMP accumulation and hyperactivation of HCN4 channels during stress [24, 56]. Conversely, CIRP deficiency exacerbates isoproterenol‐induced tachycardia through PDE4 downregulation, illustrating its critical role in maintaining cAMP homeostasis [24]. Known for its role in chromosomal cohesion, SGO1 has a non‐canonical function in the SAN [25]. SGO1 contributes to the stability of the membrane clock by physically interacting with and stabilising HCN4 channel complexes, enhancing the diastolic depolarisation rate by amplifying the hyperpolarisation‐activated current *I*_f_ [25]. Disruption of SGO1‐HCN4 interaction perturbs the kinetics of the pacemaker current, linking genomic stability proteins to the precision of the membrane clock [57, 58]. Notably, GLP‐1, an incretin hormone with pleiotropic cardiovascular effects, has been implicated in SAN regulation. GLP‐1 receptor (GLP‐1R) activation on PCs enhances *I*_f_ current density via cAMP/PKA‐dependent phosphorylation of HCN4, augmenting diastolic depolarisation rate. Concurrently, GLP‐1R signalling appears to modulate mitochondrial energetics to sustain Ca^2+^ cycling efficiency, particularly under metabolic stress [26]. However, chronic GLP‐1R overstimulation may paradoxically destabilise SAN automaticity by exacerbating cAMP/PKA signalling.

Collectively, the emerging roles of CIRP, SGO1, and GLP‐1 underscore a multi‐faceted regulatory network that supplements the coupled‐clock theory. Although acting through distinct mechanisms, CIRP mediates stress‐induced pacemaker adaptation, SGO1 safeguards chromosomal stability during cardiomyocyte cycling, and GLP‐1 modulates energy substrate utilisation, these novel modulators collectively ensure the robustness, stability, and metabolic flexibility of SAN automaticity. Their integration illustrates that the SAN pacemaker activity is not only governed by the membrane clock and calcium clock but is also profoundly influenced by cellular stress, genomic integrity, and systemic metabolic status.

## 5 Conclusions and Future Perspective

In conclusion, accumulating evidence suggests cardiac pacemaking is potentially regulated by sophisticated networks within the SAN, involving pacemaker cells microenvironmental crosstalk [19], glutamatergic signalling [21, 22], and novel molecular interactions [24, 25, 26]. Central to this complexity is the SAN's ability to integrate multiple mechanisms, exhibiting both synergistic cooperation and compensatory plasticity to ensure robust chronotropic function under diverse physiological and pathological conditions. Specifically, the fibroblast‐driven metabolic reprogramming via Aldoc enhances glycolytic flux, providing essential ATP for pacemaker activity. Concurrently, glutamatergic signalling, mediated through mitochondrial EAAT1 and ROS, amplifies LCRs, thereby synchronising the calcium and membrane clocks [21]. Furthermore, molecular regulators such as CIRP, SGO1, and GLP‐1 modulate cAMP/PKA and HCN4 channel activities, ensuring stability and responsiveness of SAN automaticity under stress [24, 25, 26].

Dysregulation of these pathways may underlie SAN pathology. Fibrotic remodelling in heart failure can disrupt SAN electrophysiology, leading to conduction abnormalities [45], while experimental disruption of EAAT1, SGO1 (e.g., K23E mutation impairing HCN4 cell‐surface expression), or CIRP (with knockout increasing beating rate under stress) disturb firing rates and pacemaker activity [21, 24, 25]. These regulatory mechanisms likely operate within specialised membrane microdomains such as caveolae, which is known to organise key pacemaker proteins and signalling complexes [59, 60]. For instance, the GLP‐1R has been shown to co‐localise and functionally interact with caveolin‐3, and this interaction is essential for the cardioprotective effects of the GLP‐1R agonist against ischemia/reperfusion injury and receptor trafficking [61]. Similarly, caveolin‐1 regulates the delivery and endocytosis of glutamate transporter, underscoring its role as a scaffolding and trafficking organiser [62]. While direct evidence for GLP‐1R and other novel modulators localisation in SAN caveolae is still lacking. Therapeutically, GLP‐1 receptor agonists, which exert cardioprotective effects without elevating atrial fibrillation risk, represent promising candidates for cardioprotection [63].

Future research should elucidate whether GLP‐1R and related regulators are organised within caveolar microdomains of the SAN and how their dysregulation contributes to SAN dysfunction. Particular attention should be paid to the translational applicability of modulating glutamatergic signalling, metabolic coupling, and molecular stabilisers such as SGO1 and CIRP in preclinical models of SAN disease. By linking molecular crosstalk to therapeutic innovation, we can advance the understanding of SAN plasticity and develop targeted interventions for SAN‐related arrhythmias.

## Author Contributions

H.L. and Y.C. wrote the manuscript and prepared figures. X.S. and H.L. revised and funded the project. All authors reviewed and revised the manuscript and figures.

## Conflicts of Interest

The authors declare no conflicts of interest.

## Data Availability

Data sharing not applicable to this article as no datasets were generated or analysed during the current study.
